# Refined scan protocol for the evaluation of pulmonary perfusion standardized image quality and reduced radiation dose in dynamic chest radiography

**DOI:** 10.1002/acm2.14222

**Published:** 2023-11-27

**Authors:** Kenta Takakura, Yuzo Yamasaki, Taku Kuramoto, Satoshi Yoshidome, Tomoyuki Hida, Takeshi Kamitani, Hideki Yoshikawa, Toyoyuki Kato, Kousei Ishigami

**Affiliations:** ^1^ Division of Radiology Department of Medical Technology Kyushu University Hospital Fukuoka Japan; ^2^ Department of Clinical Radiology Graduate School of Medical Sciences Kyushu University Fukuoka Japan; ^3^ Department of Radiological Technology Faculty of Health Sciences Kobe Tokiwa University Kobe Japan; ^4^ Department of Radiology Onga Nakama Medical Association Onga Hospital Fukuoka Japan

**Keywords:** body mass index, dynamic chest radiography, pulmonary perfusion, scan protocol

## Abstract

**Purpose:**

Dynamic chest radiography (DCR) is a novel imaging technique used to noninvasively evaluate pulmonary perfusion. However, the standard DCR protocol, which is roughly adapted to the patient's body size, occasionally causes over‐ or underexposure, which could influence clinical evaluation. Therefore, we proposed a refined protocol by increasing the number of patient body mass index (BMI) categories from three to seven groups and verified its usefulness by comparing the image sensitivity indicators (S‐values) and entrance surface doses (ESDs) of the conventional protocol with those of our refined protocol.

**Methods:**

This retrospective observational study included 388 datasets (standing position, 224; supine position, 164) for the conventional protocol (December 2019–April 2021) and 336 datasets (standing position, 233; supine position, 103) for the refined protocol (June–November 2021). The conventional protocol (BMI‐3 protocol) divided the patients into three BMI groups (BMI < 17, 17≤BMI < 25, and BMI ≥ 25 kg/m^2^), whereas the refined protocol (BMI‐7 protocol) divided the patients into seven BMI groups (BMI < 17, 17 ≤ BMI < 20, 20 ≤ BMI < 23, 23 ≤ BMI < 26, 26 ≤ BMI < 29, 29 ≤ BMI < 32, and BMI ≥ 32 kg/m^2^). The coefficients of variation (CVs) for the S‐values and ESDs acquired using the two protocols were compared.

**Results:**

The CVs of the S‐values in the BMI‐7 protocol group were significantly lower than those in the BMI‐3 protocol group for the standing (28.8% vs. 16.7%; *p* < 0.01) and supine (24.5% vs. 17.7%; *p* < 0.01) positions. The ESDs of patients scanned using the BMI‐7 protocol were significantly lower than those scanned using the BMI‐3 protocol in the standing (1.3 vs. 1.1 mGy; *p* < 0.01) and supine positions (2.5 vs. 1.6 mGy; *p* < 0.01), although the mean BMI of the two groups were similar.

**Conclusion:**

We introduced the BMI‐7 protocol and demonstrated its standardized image quality and reduced radiation exposure in patients undergoing DCR.

## 1 INTRODUCTION

Dynamic chest radiography (DCR) is a novel functional x‐ray imaging technique capable of capturing sequential chest radiographs at an imaging rate of 15 frames per second (fps) using a synergistic fusion between a flat‐panel detector (FPD) and an x‐ray generator. DCR images acquired during breath‐hold can accomplish the non‐invasive visualization of pulmonary perfusion changes in the form of alterations in pixel values within lung density, without the need for contrast media or radioisotopes.^1^, ^2^, ^3^ The clinical potential of DCR has been recognized in research on several pulmonary diseases.^4^, ^5^, ^6^, ^7^, ^8^, ^9^, ^10^, ^11^ It can be performed in standard radiography rooms with a total incident entrance surface dose (ESD) less than that of two conventional plain chest radiographs (frontal and lateral).^12^ The inherent non‐invasiveness and minimal radiation exposure of DCR, combined with its availability, profoundly enhance its clinical utility and solidify its position as an exceptional and valuable alternative among various imaging modalities. However, as DCR is a recently introduced imaging technique, the scan parameters have not been refined. Currently, the widely used standard protocol provided by manufacturers, which broadly classifies patient body size as small, medium, or large, can occasionally cause over‐ or underexposure as the settings are not individualized for each patient's specific body size. In case of overexposure, an unnecessary increase in the radiation dose is concerning. Moreover, underexposure affects the pixel values of the image, and the color mapping image after pulmonary perfusion analysis may change, potentially leading to misdiagnosis. Determining over‐ or underexposure by the appearance of the radiograph is difficult because the digital radiography (DR) system automatically optimizes the image gradation. In addition, several studies have analyzed pixel values and their temporal changes in sequential DCR images and found that they can provide important information on hemodynamics and pulmonary function.^7^, ^13^ However, variations in body size among participant groups could influence x‐ray translucency and have a significant impact on the obtained parameters. To overcome these problems, establishing a more subdivided protocol based on body mass index (BMI) with standardized image quality is necessary. The image sensitivity indicator in DR systems can provide feedback to users regarding the dose incident on the detector.^14^ The image sensitivity indicator is used as a guide in clinical practice to avoid unnecessarily high or extremely low doses because of its strong correlation with the dose incident on the detector.^15^ Similarly, establishing a refined scan protocol for DCR using an image sensitivity indicator is clinically required to manage the radiation dose in consideration of the patient's body size and to standardize the image quality. Therefore, we proposed a refined protocol by increasing the BMI categories from three to seven and verified its usefulness by comparing the image sensitivity indicators and ESDs of the conventional protocol with those of our refined protocol.

## 2 METHODS

This retrospective observational study was approved by our hospital's institutional review board, and the requirement for informed consent was waived owing to the retrospective nature of the study.

### 2.1 Study participants

We identified 726 patients who underwent DCR to evaluate pulmonary perfusion at our institution from December 2019–November 2021, including 389 datasets (standing, 224; supine, 165) from December 2019–April 2021 for the conventional protocol and 337 datasets (standing, 234; supine, 103) from June–November 2021 for the refined protocol. Patients who were unable to hold their breath and patients with poor image quality were excluded (*n* = 2). Eventually, 388 datasets (standing, 224; supine, 164) from patients scanned with the conventional protocol from December 2019–April 2021 and 336 datasets (standing, 233; supine, 103) from patients scanned with the refined protocol from June–November 2021 were analyzed in this study. The patient characteristics for each protocol are shown in Table 1. To compare the differences in sex and BMI between the patient groups for each protocol, *p*‐values were calculated in the standing and supine positions, respectively. Between‐sex differences were evaluated using the chi‐square test. A two‐tailed Student's *t*‐test was used to compare BMI groups between the two protocols.

**TABLE 1:** Patient characteristics.

		BMI‐3 protocol (*n* = 388)	BMI‐7 protocol (*n* = 336)	*p*‐value
Standing	Male/Female	98 (44%)/126 (56%)	129 (55%)/104 (45%)	0.01
Supine	Male /Female	71 (43%)/93 (57%)	54 (52%)/49 (48%)	0.14
BMI (kg/m^2^)	Standing	23 ± 4, range (12−39)	23 ± 4, range (14−39)	0.37
	Supine	23 ± 4, range (12−37)	23 ± 4, range (14−39)	0.65

*Note*: Sex data are presented as the number (%) of patients.

BMI data are expressed as the mean ± standard deviation (minimum to maximum).

Abbreviation: BMI, body mass index.

### 2.2 Scan protocols

A dynamic FPD imaging system comprising an indirect conversion FPD (AeroDRfine, Konica Minolta, Inc., Tokyo, Japan), an x‐ray tube (0.6/1.2P324DK‐85, Shimadzu Corporation, Kyoto, Japan), and an x‐ray generator (UD150B‐40, Shimadzu Corporation) were used in this study. Automatic exposure control is not included in this dynamic imaging system. The matrix size of the images was 1024 × 1024 pixels, pixel size was 400 × 400 μm, total image area was 424.8 × 424.8 mm, and the gray‐scale range was 16 bits. The pixel size in dynamic imaging is four times larger than that of static imaging (100 μm) because 4 × 4 pixels are treated as one signal by binning. Image processing was performed using the PH2‐MODE algorithm to visualize lung perfusion on an x‐ray dynamic image analysis workstation (KINOSIS version 1.2, Konica Minolta, Inc.). PH2‐MODE is a pulmonary perfusion analysis method based on temporal changes in pixel values during the cardiac cycle (Appendix A).^8^ First, the end‐diastolic phase is estimated from the temporal change in the heart and defined as the reference phase. Then, temporal changes in pixel values from the reference phase in each pixel in the lung is calculated and visualized as pulmonary perfusion. This analysis method helps to evaluate the distribution of pulmonary perfusion in each lung area in a semi‐quantitative manner.

Two different protocols were applied in this study: the first classified BMI into three groups (BMI‐3 protocol) and the second into seven groups (BMI‐7 protocol) (Table 2). In the BMI‐3 protocol, BMI was classified as BMI < 17, 17 ≤ BMI < 25, and BMI ≥ 25 kg/m^2^, and the radiographic exposure factors were determined for each group. In the BMI‐7 protocol, BMI was classified into seven groups as follows: BMI < 17, 17 ≤ BMI < 20, 20 ≤ BMI < 23, 23 ≤ BMI < 26, 26≤BMI < 29, 29 ≤ BMI < 32, and BMI ≥ 32; the tube current‐time product (mAs) was adjusted according to the BMI. The BMI‐3 protocol was based on the protocol recommended by the manufacturer, whereas the BMI‐7 protocol is the refined protocol to be tested in this study. DCR is a new technique, and the standard image sensitivity indicators are different from those of conventional plain chest radiography, although they have not been studied in detail. Based on our clinical experience, the number of clinically acceptable target image sensitivity indicators was set at 3000 according to the consensus of the radiologic technologists (K.T., T.K., and H.Y.) and radiologists (Y.Y., T.K., and T.H.). Then, in the preliminary experiments, we investigated the relationship between mAs and the image sensitivity indicator using a chest phantom (N1 “LUNGMAN,” Kyoto Kagaku Co., Ltd., Kyoto, Japan). Additionally, the radiographic exposure factors that could obtain target image sensitivity indicators were examined, and the BMI‐7 protocol was adopted (Appendix B). The imaging rate was set to 15 fps for all radiographic conditions. All patients were instructed to breathe deeply with inspiration and hold their breath for 7 s to evaluate pulmonary perfusion. This system automatically controls the x‐ray exposure time from start to finish over a 7‐s period.

**TABLE 2:** BMI‐3 and BMI‐7 protocols.

	BMI‐3 protocol			BMI‐7 protocol		
Position	BMI (kg/m^2^)	kV	mAs	BMI (kg/m^2^)	kV	mAs
Standing SID: 2 m additional filter: 0.2 mm Cu	BMI < 17	85	0.64	BMI < 17	85	0.64
				17 ≤ BMI < 20	100	0.4
				20 ≤ BMI < 23	100	0.504
	17 ≤ BMI < 25	100	0.64	23 ≤ BMI < 26	100	0.568
				26 ≤ BMI < 29	110	0.55
				29 ≤ BMI < 32	110	0.625
	BMI ≥25	110	0.625	BMI ≥32	110	0.9
Supine SID: 1.5 m additional filter: 0.3 mm Cu	BMI < 17	85	1	BMI < 17	85	0.55
				17 ≤ BMI < 20	95	0.45
				20 ≤ BMI < 23	95	0.625
	17 ≤ BMI < 25	95	1	23 ≤ BMI < 26	95	0.8
				26 ≤ BMI < 29	95	1
				29 ≤ BMI < 32	95	1.125
	BMI ≥25	95	1.575	BMI ≥32	95	1.25

Abbreviations: BMI, body mass index; kV, tube voltage; mAs: tube current‐time product; SID, source‐to‐image distance.

### 2.3 Image sensitivity indicator

For DCR images, radiologic technologists recorded the scan protocols, patient's position, BMI, body thickness, and the image sensitivity indicator of the DCR image. Body thickness was measured at the level of the center of the patient's sternum using calipers. The image sensitivity indicator of the system (S‐value) has been described as the most important tool for image quality control while maintaining the minimum patient dose.^15^ For the imaging system used in this study, the S‐value was defined by the following equation:

(1) $$S=200\times \left(1\left[\mathrm{mR}\right]/D\left[\mathrm{mR}\right]\right)$$

where D is the actual dose incident on the detector. For a dose incident on the detector of 1 mR at 80 kVp, the S‐value is 200.^16^ The half‐value layer at 80 kVp as the calibration condition was 2.7 mmAl. Equation (1) is applied at all tube voltages, and lung fields and mediastinal structures are automatically extracted from the acquired images and calculated for a set region of interest. The S‐value is inversely proportional to the dose incident on the detector.^14^ When the same patient is imaged using the same beam quality and positioning and the mAs are doubled, the S‐value will be approximately halved.^16^ In other words, although indirectly, a correlation between the S‐value and patient dose exists. The S‐value was used as an indicator to optimize the dose while maintaining image quality. The S‐values obtained under the BMI‐3 and BMI‐7 protocols were divided by BMI group, and the means and coefficients of variation (CVs) were calculated. The mean CVs for the overall BMI groups were compared in the standing and supine positions between protocols.

### 2.4 Measurement of ESD

The total ESD of DCR is calculated by multiplying the ESD per pulse (mGy/pulse) with the imaging rate (fps) and imaging period (s). The total ESD was measured under the conditions of 15 fps and 7 s. The ESD, including backscatter, was measured using an ionization chamber dosimeter (Model 9095; Radcal Corporation, Monrovia, CA, USA) on a water‐equivalent phantom (Solid Water, Gammex‐RMI, Middleton, WI, USA) with the same thickness as the patient's actual body thickness. Because the scan protocols were BMI‐based, the thickness of the phantom was determined from the patient's BMI and clinically obtained thickness data. In the BMI‐3 protocol, the mean body thicknesses for each BMI group were 16, 18, and 22 cm, respectively. In the BMI‐7 protocol group, the mean body thicknesses for each BMI group were 15, 16, 18, 20, 21, 22, and 24 cm, respectively. The mean body thickness in each BMI subgroup in the BMI‐7 protocol group was used as the phantom thickness, and the ESD was measured in each protocol.

The ESD measured at the mean body thickness in each BMI subgroup in the BMI‐3 and BMI‐7 protocols was used as the mean ESD of patients who actually underwent DCR; the mean ESDs for the overall BMI groups were compared in the standing and supine positions between protocols.

### 2.5 Changes in the color mapping image with dose reduction simulation

We used simulation to confirm the changes in pulmonary perfusion in the color mapping images according to the difference in S‐values.

The original images of a patient with chronic thromboembolic pulmonary hypertension (age, 79 years; BMI, 21.3 kg/m^2^) were simulated using dose levels corresponding to S‐values of 2000, 3000, 4000, 5000, and 6000 by adding white noise with a specific standard deviation to the original dynamic images.^17^ First, the signal values in the lung fields of the original images were scaled to the signal value intensity corresponding to each S‐value. White noise was then added to the scaled image. This white noise comprised quantum noise with a constant noise power spectrum that causes uniform pixel value fluctuations throughout the image. A linear relationship exists between the variance of the white noise and the signal value (i.e., pixel value) in the lung field. Therefore, a specific standard deviation corresponding to the level of each S‐value was added to the original dynamic images using normally distributed random numbers. The pixel value changes in the region of interest placed in the left ventricle were Fourier transformed, and the pulse‐related frequencies in the heart were calculated. The original and simulated low‐dose dynamic images were bandpass filtered at the calculated frequency of 0.1 Hz, and the signal values of only the cardiac cycle component in each lung pixel were extracted. Color mapping images were produced using PH2‐MODE, which is commonly used in clinical practice to visualize pulmonary perfusion.

### 2.6 Statistical analysis

S‐values for each BMI group are expressed as the mean ± standard deviation. The variation in S‐values for both scan protocols was calculated as the CV and statistically compared using the F‐test. The CV is calculated by dividing the standard deviation by the mean and then multiplying by 100. The Welch t‐test was used to identify statistically significant differences in ESDs between the BMI‐3 and BMI‐7 protocols. All statistical analyses were performed using Microsoft Excel 2019 (Microsoft Corp., Redmond, WA, USA). Statistical significance was set at *p* < 0.05.

## 3 RESULTS

### 3.1 S‐values under the BMI‐3 versus BMI‐7 protocol

**FIGURE 1:**
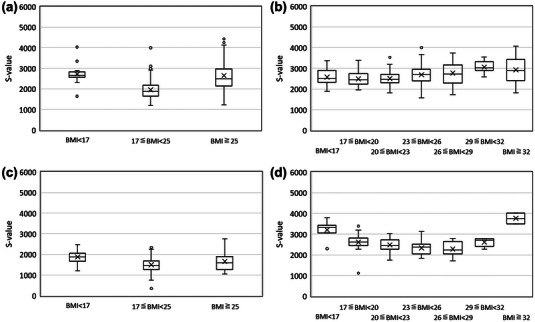
Distribution of S‐values obtained under scan protocols in standing and supine positions. (a) The BMI‐3 and (b) BMI‐7 protocols in the standing position, (c) BMI‐3, and (d) BMI‐7 protocols in the supine position. The upper and lower edges of each box represent 75% and 25%, respectively, with the centerline representing the median. The means are indicated by crosses and outliers by circles. BMI, body mass index.

Figure 1 depicts box plots of S‐values and BMIs of the patients under the BMI‐3 and BMI‐7 protocols in the standing and supine positions. The median S‐values for the standing position varied widely in the BMI‐3 protocol group (Figure 1a). In contrast, the BMI‐7 protocol group exhibited a constant median S‐value ranging from 2442 to 3024, regardless of the patient's BMI (Figure 1b). The median S‐values for the supine position ranged from 1457 to 1862 in the BMI‐3 protocol group (Figure 1c). The BMI‐7 protocol group exhibited constant median S‐values ranging from 2247 to 2713 for BMI groups except for the BMI < 17 and BMI ≥ 32 kg/m^2^ groups (Figure 1d).

Table 3 shows the S‐values and CVs of patients who underwent DCR under the BMI‐3 and BMI‐7 protocols. The CVs for all patients under the BMI‐3 protocol were significantly higher than those of patients under the BMI‐7 protocol in the standing (28.8 % vs. 16.7 %, *p* < 0.01) and supine (24.5 % vs. 17.7 %, *p* < 0.01) positions.

**TABLE 3:** S‐values and ESDs in patients who underwent DCR under the BMI‐3 and BMI‐7 protocols.

	BMI‐3 protocol					BMI‐7 protocol				
Position	BMI (kg/m^2^)	S‐value	CV (%)	ESD (mGy)	n	BMI (kg/m^2^)	S‐value	CV (%)	ESD (mGy)	n
Standing	BMI < 17	2725 ± 546	20.0	0.7	(13)	BMI < 17	2577 ± 403	15.7	0.7	(12)
						17 ≤ BMI < 20	2489 ± 329	13.2	0.8	(44)
						20 ≤ BMI < 23	2506 ± 337	13.4	1.0	(65)
	17 ≤ BMI < 25	1957 ± 460	23.5	1.2	(157)	23 ≤ BMI < 26	2698 ± 446	16.6	1.2	(66)
						26 ≤ BMI < 29	2770 ± 558	20.2	1.3	(26)
	BMI ≥25	2652 ± 712	26.8	1.5	(54)	29 ≤ BMI < 32	3064 ± 278	9.1	1.5	(12)
						BMI ≥32	2928 ± 723	24.7	2.2	(8)
	Overall	2169 ± 625	28.8	1.3	(224)	Overall	2513 ± 383	16.7	1.1	(233)
Supine	BMI < 17	1877 ± 341	18.6	1.4	(11)	BMI < 17	3207 ± 467	14.6	0.8	(7)
						17 ≤ BMI < 20	2618 ± 522	19.9	1.0	(15)
	17 ≤ BMI < 25	1527 ± 460	30.1	2.2	(113)	20 ≤ BMI < 23	2480 ± 278	11.2	1.4	(31)
						23 ≤ BMI < 26	2339 ± 325	13.9	1.9	(34)
						26 ≤ BMI < 29	2283 ± 360	15.8	2.4	(8)
	BMI ≥25	1659 ± 459	27.7	3.8	(40)	29 ≤ BMI < 32	2618 ± 207	7.9	2.7	(6)
						BMI ≥32	3766 ± 371	9.8	3.1	(2)
	Overall	1562 ± 383	24.5	2.5	(164)	Overall	2521 ± 447	17.7	1.6	(103)

Data are expressed as the mean ± standard deviation.

BMI, body mass index, CV, coefficient of variation; ESD, entrance surface dose; DCR, dynamic chest radiography.

The CV is the standard deviation divided by the mean, multiplied by 100.

### 3.2 ESDs in the BMI‐3 versus BMI‐7 protocol

**FIGURE 2:**
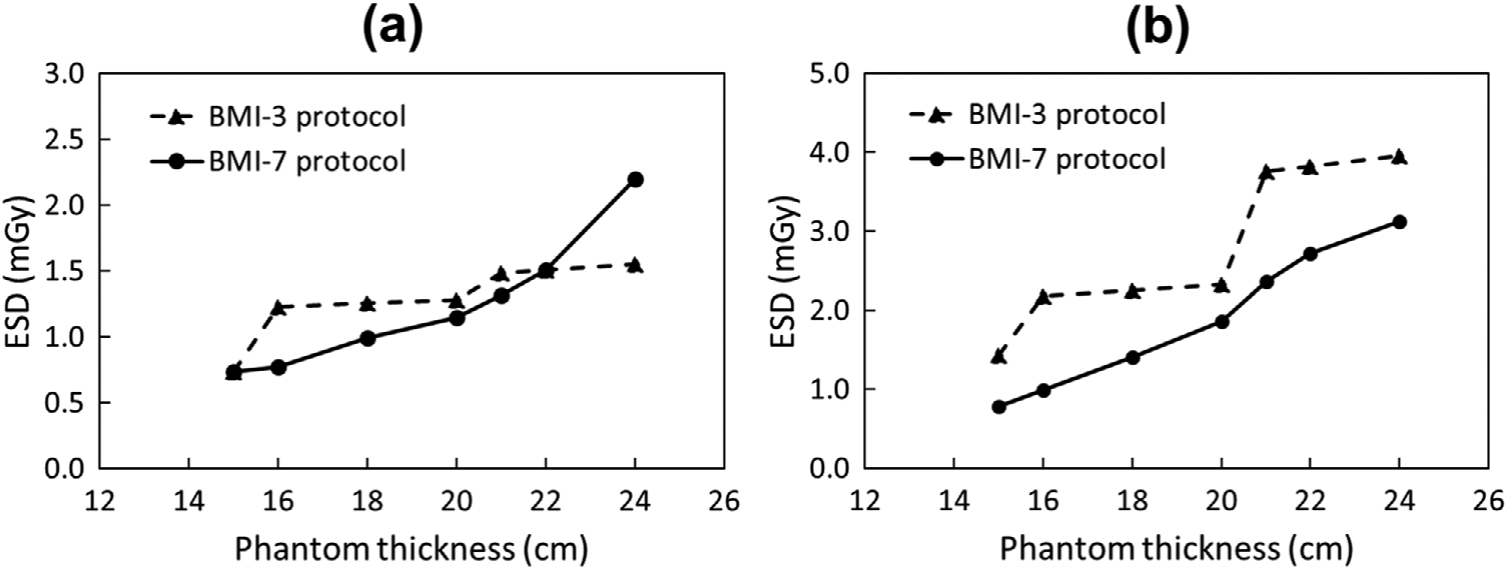
Results of the entrance surface dose measured with the BMI‐3 and 7 protocols in a water‐equivalent phantom. (a) The standing, and (b) supine positions. BMI, body mass index; ESD, entrance surface dose.

Figure 2 shows the ESDs measured with the BMI‐3 and BMI‐7 protocols in a water‐equivalent phantom. The ESDs in the BMI‐7 protocol were reduced by a maximum of 37.0% and 54.7% in the standing and supine positions, respectively, with a phantom thickness of 16 cm. The ESDs in the BMI‐7 protocol were lower than those in the BMI‐3 protocol, except for those with a phantom thickness of 24 cm.

Table 3 shows the ESDs in patients who underwent DCR under the BMI‐3 and BMI‐7 protocols. The mean ESDs for all BMI groups scanned using the BMI‐7 protocol were significantly lower than those of groups scanned using the BMI‐3 protocol in the standing (1.3 vs. 1.1 mGy; *p* < 0.01) and supine (2.5 vs. 1.6 mGy; *p* < 0.01) positions (Table 3), whereas no significant differences were observed in BMI subgroups between the BMI‐3 and BMI‐7 protocol groups (23 ± 4 vs. 23 ± 4; *p* = 0.37−0.65) (Table 1).

### 3.3 Differences in original versus simulation images with multiple S‐values

**FIGURE 3:**
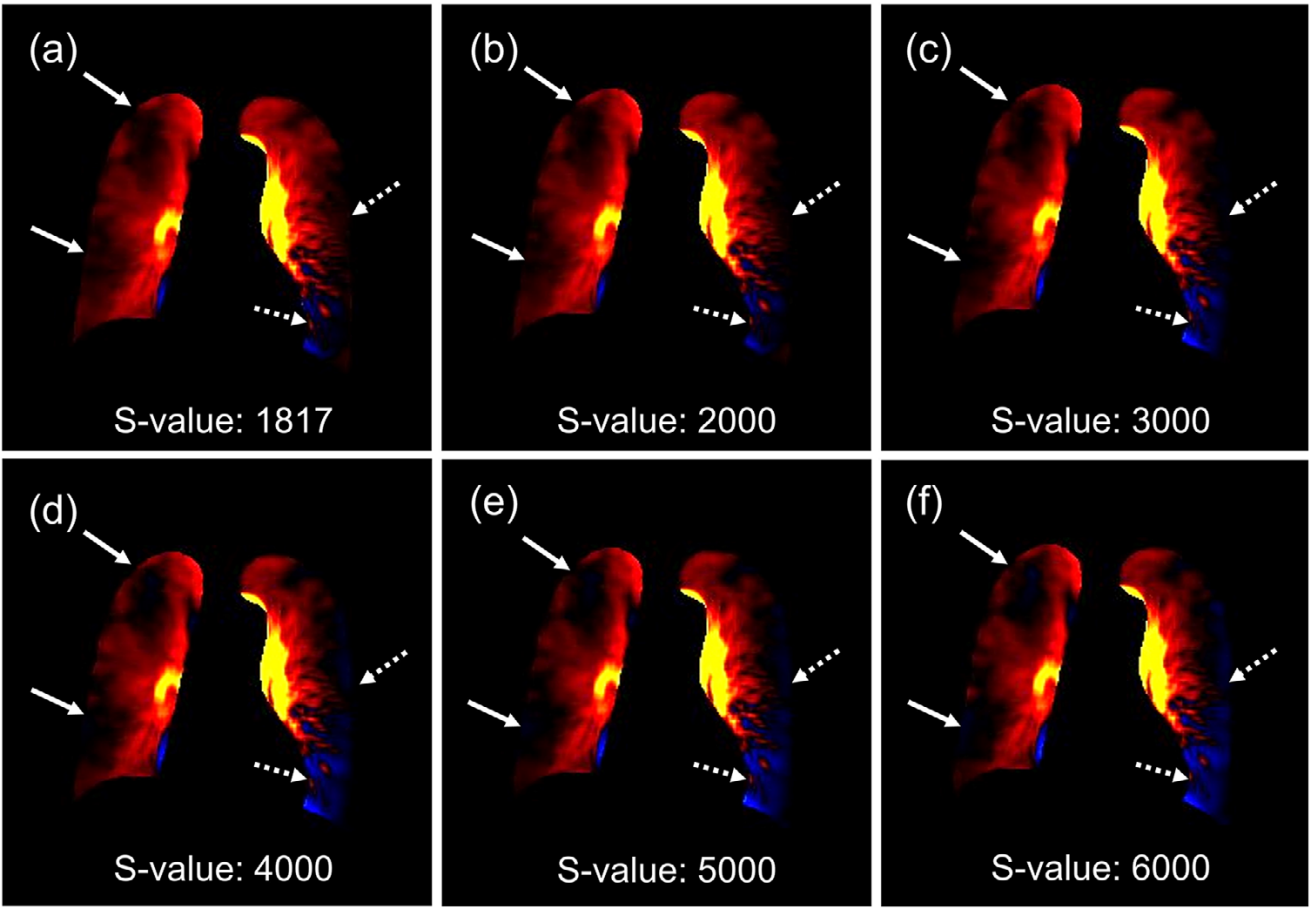
Pulmonary perfusion color mapping images of various S‐values. S‐values of (a) 1817, (b) 2000, (c) 3000, (d) 4000, (e) 5000, and (f) 6000 in a 79‐year‐old woman with chronic thromboembolic pulmonary hypertension. The pulmonary perfusion defects were greater with increasing S‐values (arrows). Furthermore, a reduction in perfusion is observed at the outer edge of the left lung and at the edge of the heart (dotted arrows).

Figure 3 shows the pulmonary perfusion color mapping images with various S‐values for the same patient. The original and simulated color mapping images show multiple perfusion defects in both lung fields. The pulmonary perfusion defects were greater with increasing S‐values.

## 4 DISCUSSION

Herein, we compared the S‐values and ESDs of DCR images obtained under the BMI‐3 and BMI‐7 protocols. We found that the BMI‐7 protocol exhibited reduced variability in S‐values and lower ESDs across most BMI subgroups.

Under the BMI‐3 protocol, the S‐values obtained for the 17≤BMI < 25 and BMI ≥ 25 groups varied widely between standing and supine positions (Figure 1a and 1c). Moreover, the variability in the S‐values increased as the BMI increased. BMI can be used as a surrogate for body thickness, and patients with higher BMIs also have increased chest thickness.^18^ Therefore, under the same scan protocols, the x‐ray dose transmitted through the body is lower, resulting in a lower dose incident on the detector for patients with higher BMIs,^19^ leading to increased S‐values. Furthermore, within the BMI‐3 protocol, the wider range of BMI classifications resulted in greater variations in chest thickness, leading to increased variability in the dose incident on the detector within each BMI group. In contrast, under the BMI‐7 protocol, the median S‐values were distributed within a narrow range in the standing and supine positions, except for BMI < 17 and BMI ≥ 32 in the supine position (Figures 1b and 1d). The exceptions observed in the BMI < 17 and BMI ≥ 32 groups can be attributed to the distribution bias resulting from the small number of patients in these BMI groups. The median S‐value was higher in the supine position among patients with a BMI < 17 kg/m^2^ because half of the patients had a BMI ranging from 16 to 17 kg/m^2^. One of the two patients in the BMI ≥ 32 group had a very high BMI (39 kg/m^2^). The BMI‐7 protocol did not affect the interquartile range but had the advantage of converging the median S‐value to the target value, shortening the maximum and minimum range and reducing outliers.

The BMI‐7 protocol was associated with lower doses compared with the BMI‐3 protocol for all BMI groups, except for the BMI ≥ 32 group in the standing position, while achieving less variability in S‐values. However, ESDs in the standing position for the BMI ≥ 32 group under the BMI‐7 protocol were higher than those under the BMI‐3 protocol (2.2 vs. 1.5 mGy). The total recommended DCR dose is < 1.9 mGy^12^ for two conventional plain chest radiographs (frontal and lateral).^1^ Under the BMI‐7 protocol, ESDs increased step‐by‐step with body thickness (Figure 2). Therefore, radiation doses > 1.9 mGy were required to maintain the appropriate S‐values in the higher BMI groups. This finding is important when considering image quality maintenance in groups with large body sizes.

Our results provide clinical benefits in that decreasing the S‐value variability of the images leads to a decreased frequency of images with unexpectedly high S‐values. As shown in Figure 3, the areas of perfusion defects in the right upper and lower lungs increased as the S‐value increased. Furthermore, a reduction in perfusion was observed at the outer edge of the left lung and the edge of the heart. Similar results are expected in cases with high S‐values and may lead to over‐ or misdiagnosis of pulmonary embolism. However, if the S‐value is too low, the appearance of the color mapping image will not change much, and an unnecessary increase in the radiation dose is concerning. Therefore, setting the scan protocol according to the patient's size is essential. Moreover, standardized S‐values could have a beneficial impact on the semi‐quantitative assessment of pulmonary perfusion for sequential assessments of diseases and the evaluation of treatment efficacy in the future.

This study has some limitations. First, we did not refer to x‐ray sensitivity indicators for systems developed by other manufacturers. Definitions of x‐ray sensitivity indicators in DR systems vary among manufacturers, and the algorithms are complicated. However, because the system we used in this study does not support the exposure index, we used S‐values as an x‐ray sensitivity indicator. Considering the exposure index is an internationally accepted standard, further studies, including the exposure index, may lead to further standardization of image acquisition in the future. Additionally, S‐values vary with factors such as the beam quality, variation in the output of the tube of each imaging system, and system sensitivity, making it difficult to estimate the dose incident on the detector accurately. Therefore, investigating the relationship between S‐values and the dose incident on the detector for each imaging system is necessary and may be used as an indicator to avoid unnecessarily high or extremely low doses. Second, this study lacks information on the S‐value at which pulmonary perfusion may be most appropriate. Further studies are needed to investigate which S‐values are adequate for the clinical assessment of pulmonary diseases, especially pulmonary embolism. Third, a common tube voltage was not used for each protocol. Therefore, the x‐ray incident spectrum on the patient differed in each protocol, potentially affecting the obtained images. However, this study focused only on demonstrating the utility of a refined protocol with a more detailed classification of BMI. Further studies are necessary to determine the effect of tube voltage on pulmonary perfusion in color mapping images. Fourth, although a linear relationship exists between BMI and chest thickness,^18^ variations in chest thickness exist even among patients with the same BMI. However, the actual measurement of chest thickness has a large margin of error due to differences among measures. However, BMI can be easily calculated from a patient's height and weight and used as an objective body size index.

## 5 CONCLUSION

We proposed the BMI‐7 protocol and demonstrated its standardized image quality and reduced radiation exposure in patients undergoing DCR. This protocol can be used to evaluate pulmonary perfusion more precisely than the BMI‐3 protocol. Image acquisition under the refined BMI‐7 protocol, which includes a more detailed classification of BMI, is recommended for DCR.

## AUTHOR CONTRIBUTIONS

Kenta Takakura, Yuzo Yamasaki, Taku Kuramoto, Satoshi Yoshidome, Hideki Yoshikawa: Conceptualization, Methodology, Validation,Writing‐Review and Editing, Visualization. Kenta Takakura, Satoshi Yoshidome: Formal analysis. Kenta Takakura: Investigation, Data Curation. All authors.: Writing‐Original Draft, Supervision, Project administration.

## CONFLICT OF INTERESTS STATEMENT

Yuzo Yamasaki, Tomoyuki Hida, Takeshi Kamitani, and Kousei Ishigami received the research grant from Konica Minolta. The other authors have no financial support related to this work. The authors have received technical support for image creation from KONICA MINOLTA, Japan.

## Data Availability

The data that support the findings of this study are available from the corresponding author upon reasonable request.

## APPENDIX A

### A.1

PH2‐MODE analysis method can be used to identify pulmonary perfusion in dynamic chest radiography images and create a lung perfusion map.^8^

(A) The temporal changes in pixel values for each pixel is calculated from sequential images.
(B) A bandpass filter is applied to extract cardiac cycle‐related signals.
(C) The end‐diastolic and end‐systolic phases are automatically estimated from the pixel value changes in the heart. The timing of the highest pixel value, which represents the maximum blood volume in the heart, is defined as the end‐systolic phase.
(D) The temporal changes in the pixel values from the end‐diastolic phase are color‐coded and visualized as dynamic perfusion images. Low blood volume is indicated in blue, and high blood volume is indicated in red. Black indicates no change in the interval.
(E) Finally, the sequential perfusion images are obtained.
(F) The maximum intensity projection image in the lung, which is determined by semiautomatic contouring, is also created as a lung perfusion map.

## APPENDIX B

### B.1

The S‐value standardization process used in this study is described below. First, the S‐values obtained with the body mass index (BMI)−3 protocol were classified into seven BMI groups (BMI < 17, 17 ≤ BMI < 20, 20 ≤ BMI < 23, 23 ≤ BMI < 26, 26 ≤ BMI < 29, 29 ≤ BMI < 32, and BMI ≥ 32). The mean S‐values for each BMI group were calculated. Next, in a preliminary experiment, we investigated the relationship between the tube current‐time product (mAs) and S‐value using a chest phantom. S‐values were measured at different mAs values at each tube voltage (Table A1). Although the chest phantom was a standard body size, the rate of change between the mAs and S‐value remained the same for larger or smaller than standard body sizes. Finally, the exposure parameters (mAs) for each BMI group were adjusted to achieve a target S‐value of 3000 in all BMI groups.

**TABLE A1:** Results of S‐values measured with tube voltages of 100 and 110 kV (standing position) and 85 and 95 kV (supine position).

Standing				Supine			
100 kV		110 kV		85 kV		95 kV	
mAs	S‐value	mAs	S‐value	mAs	S‐value	mAs	S‐value
0.224	5264	0.3	3195	0.45	6401	0.45	3449
0.256	4651	0.35	2730	0.5	5917	0.5	3103
0.288	4082	0.4	2380	0.55	5275	0.55	2849
0.32	3714	0.45	2107	0.625	4548	0.625	2580
0.36	3260	0.5	1913	0.7	4073	0.7	2229
0.4	2946	0.55	1745	0.8	3496	0.8	2010
0.448	2627	0.625	1531	0.9	3075	0.9	1776
0.504	2306	0.7	1359	1	2842	1	1587
0.568	2061	0.8	1198	1.125	2433	1.125	1406
0.64	1833	0.9	1064	1.25	2079	1.25	1242

*Note*: mAs, tube current‐time product.
